# Hierarchical algorithms on hierarchical architectures

**DOI:** 10.1098/rsta.2019.0055

**Published:** 2020-01-20

**Authors:** D. E. Keyes, H. Ltaief, G. Turkiyyah

**Affiliations:** 1 Extreme Computing Research Center, King Abdullah University of Science and Technology, Thuwal 23955-6900, Saudi Arabia; 2 Department of Computer Science, American University of Beirut, Beirut 1107 2020, Lebanon

**Keywords:** computational linear algebra, hierarchical matrices, exascale architectures

## Abstract

A traditional goal of algorithmic optimality, squeezing out flops, has been superseded by evolution in architecture. Flops no longer serve as a reasonable proxy for all aspects of complexity. Instead, algorithms must now squeeze memory, data transfers, and synchronizations, while extra flops on locally cached data represent only small costs in time and energy. Hierarchically low-rank matrices realize a rarely achieved combination of optimal storage complexity and high-computational intensity for a wide class of formally dense linear operators that arise in applications for which exascale computers are being constructed. They may be regarded as algebraic generalizations of the fast multipole method. Methods based on these hierarchical data structures and their simpler cousins, tile low-rank matrices, are well proportioned for early exascale computer architectures, which are provisioned for high processing power relative to memory capacity and memory bandwidth. They are ushering in a renaissance of computational linear algebra. A challenge is that emerging hardware architecture possesses hierarchies of its own that do not generally align with those of the algorithm. We describe modules of a software toolkit, hierarchical computations on manycore architectures, that illustrate these features and are intended as building blocks of applications, such as matrix-free higher-order methods in optimization and large-scale spatial statistics. Some modules of this open-source project have been adopted in the software libraries of major vendors.

This article is part of a discussion meeting issue ‘Numerical algorithms for high-performance computational science’.

## 1. A renaissance in computational linear algebra

A renaissance has come to computational linear algebra in the form of hierarchically low-rank matrices (henceforth ‘H-matrices’). They are useful in a wide variety of applications leading to dense matrices, such as mechanics and electrostatics formulated in terms of Green’s functions, maximum likelihood in spatial statistics built on covariance matrices, optimization based on Hessians, and even applications of sparse matrices during which dense Schur complements are formed. Formally, dense operators are often ‘data sparse’ in the sense that their input-to-output maps can be mediated to high accuracy in far less than *n*^2^ operations, and inverted in far less than *O*(*n*^3^) operations. Indeed, the curse of dimensionality can be mitigated in these applications and others by the blessing of low rank. The emergence of algorithms exploiting hierarchical low rank over the past two decades, since the seminal work of Hackbusch [1] and Tyrtyshnikov [2], could hardly come at a more auspicious time in terms of computer architecture. A main motivation is the decreasing ratio of memory bandwidth to processing power [3] and the growing latency in clock cycles of accessing an element of deep memory, which can be a thousand or more for DRAM. Data sparsity implies that relatively small cache memories can hold relatively highly accurate representations of operators. The savings in latency from residing high on the memory hierarchy is even more important than the savings in operation count from working directly with the compressed representation.

The desire to extend computable problem sizes in spatial statistics has in recent years led researchers to consider far more drastic approximations of covariance matrices; see e.g. discussions in [4]. However, such severe approximation is not necessary to scale to larger problem sizes, since hierarchically low-rank approximations allow navigation of the accuracy-capacity trade-off in a graceful way.

Hierarchically low-rank representation predates its formulation in H-matrices; it undergirds the fast multipole method (FMM) introduced in 1986 [5]. FMM achieves reduction from quadratic to linear in the number of operations required to account for mutual particle–particle interaction by clustering particles hierarchically with distance. Memory need not be a major consideration in FMM because of the possession of an analytic expression of the Green’s function. (Storing translation operators is an optional time-space trade-off.) However, the reduction of communication from exploiting hierarchy is significant in distributed-memory implementations. As shown in [6], the same efficiencies can be achieved for H-matrices because the latter are naturally implemented with the same dual tree traversal structure as FMM, the principal difference being the need to store basis vectors for the compressed weak interactions.

Tile low-rank (henceforth ‘TLR’) matrices are straightforward generalizations of the tiled matrix data structures that have proved fruitful in migrating dense linear algebra kernels of ScaLAPACK to the many-core shared-memory environment [7,8]. We consider them a step *en route* to H-matrices from the algebraic side, just as FMM is a step *en route* to H-matrices from the analytical. TLR algorithms do not achieve the reduction to log-linear complexity of H-matrix algorithms, since they interpose only one intermediate level between scalar operations and the full discrete problem dimension, rather than a recursive hierarchy of such levels. However, TLR instantly migrates the benefits of data sparsity within a tile to the rich libraries of tile-based kernels.

## 2. Tile low-rank representation

Tile low-rank linear algebra was inspired by the block low-rank (BLR) compression of Schur complements of elliptic PDE operators in [9], which was, in turn, explored in the 2013 thesis of Weisbecker [10]. A *m* × *n* matrix *A* is practically of low rank if *A* = *UV*^*T*^ + *E*, where *U* is *m* × *k*, *V* is *n* × *k*, for *k* < *mn*/(*m* + *n*), where $||E|{|}_{2}<\epsilon$ is small enough to be neglected and rank *k* depends upon the accuracy tolerance *ϵ* [11].

In a typical TLR matrix application, *A* is partitioned in advance into blocks of uniform size related to the level of the memory hierarchy in which they should fit and/or the number of threads available on the node, with due consideration of ordering to cluster degrees of freedom with the strongest coupling along the primary and possibly other diagonals. Each tile that is believed to be a candidate for low-rank representation is then independently compressed using any of a variety of algorithms to determine an acceptable *k*, typically by considerations local to a tile. Ranks may vary across tiles; hence, the task of compression may not be load-balanced across tiles, nor may be the subsequent tasks of manipulating tiles within the context of a standard tile algorithm. The latter may require matrix-matrix multiplications, matrix-matrix additions, or the application of the inverse of a full-rank tile to other tiles. In the context of tile algorithms, this is not a major drawback because they are typically executed, as described in §4, via a task-based dynamic runtime system based on a directed acyclic graph (DAG).

**Figure 1.**
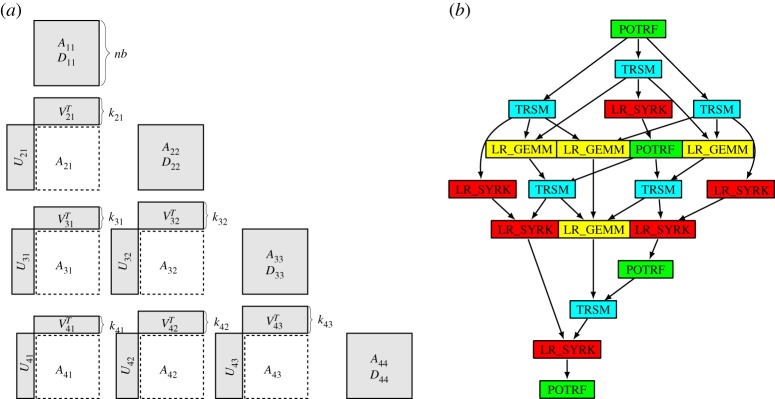
An originally dense symmetric positive definite matrix decomposed into tiles (*a*) and a DAG for its Cholesky factorization (*b*). (Online version in colour.)

For a dense symmetric positive definite matrix, a tile-based Cholesky factorization defines a sequence of diagonal block factorizations, column block scalings by diagonal block inverses, and block row multiplication and addition updates. A sample DAG for the 4 × 4 blocked symmetric matrix *A* on the left in figure 1 is shown to the right. Coloured rectangular nodes represent tasks and the arrows data dependencies. Each of the four diagonal block factorizations (POTRF, in green) is followed by block updates to its own lower subtriangle, through the last block *A*_44_, which is its own subtriangle. A tile-based Cholesky factorization is described, for instance, in [12], using, e.g. MAGMA’s [7] POTRF, with the Basic Linear Algebra Subroutines (BLAS) GEMM, TRSM, SYRK, TRMM, SYMM, SYMV and TRSV.

A TLR data structure begins with the tile decomposition of *A*. Its diagonal blocks, *D*_*ii*_, are the same as those of *A*. We then replace the off-diagonal blocks, *A*_*ij*_ for *i* > *j*, with low-rank factorizations, $U_{ij}V_{ij}^{T}$, where the factors have rank *k*_*ij*_ < *n*_*b*_, where *n*_*b*_ is the (traditionally uniform) block size. Preferred compression routines are the Randomized SVD [13], adaptive cross-approximation (ACA) [14], or QR factorization [15]. Whenever a low-rank tile is updated, it requires recompression.

**Figure 2.**
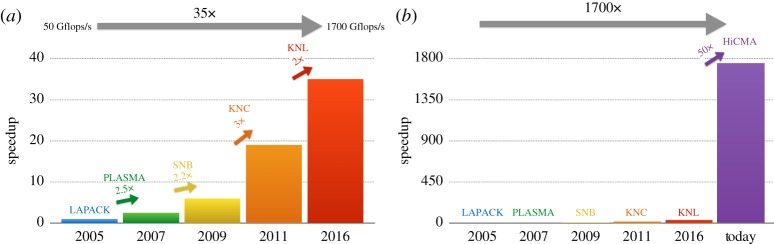
Improvements to Cholesky factorization performance from programming model and hardware, 2005–2016 (*a*) and from TLR, 2005–today (*b*; note change of vertical scale). The baseline from 2005 is an Intel 8-core Clovertown. Each successive entry reflects a DPOTRF sustained peak for matrices whose sizes grow asymptotically with the available system memory. (Online version in colour.)

The TLR technique is the latest in a string of developments in hardware, programming models, and algorithms that have led to orders of magnitude of performance improvement in the Cholesky decomposition for large symmetric positive definite matrices, an important kernel in innumerable applications, over the last 15 years. The classic LAPACK [16] algorithm for Cholesky in 2005 was a panel algorithm that achieved Level-3 BLAS performance through column blocking but suffered from artifactual over-ordering and decreasing concurrency as it proceeded down the diagonal. First-generation tile algorithms using PLASMA [7] on the same hardware in 2007 provided a factor of about 2.5 through a task-based programming model, which yields more concurrency and shortens the critical path. Over the next decade, as shown in figure 2*a*, multi- and manycore programmers followed Moore’s Law for an order of magnitude performance improvement for tile algorithms on a single processor, in an essentially ‘free lunch’ phase. As shown in figure 2*b*, the TLR technique has provided another one to two orders of magnitude of performance in the last two years, depending upon compressibility. The label atop the last bar represents the hierarchical computations on manycore architectures (HiCMA^1^) library [17], which is described herein.

**Figure 3.**
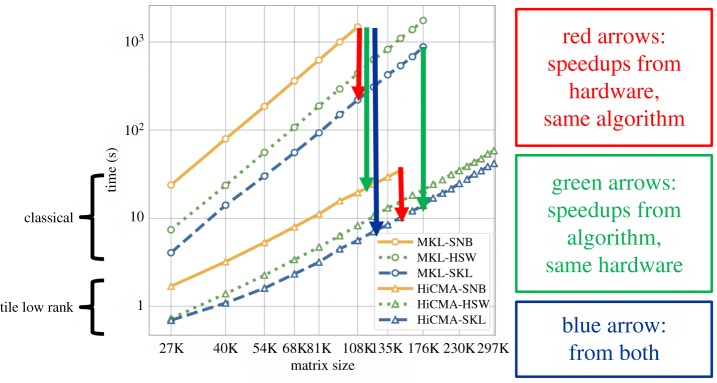
Shared-memory implementations of DPOTRF on three generations of Intel hardware and two generations of algorithms. (Online version in colour.)

Figure 3 illustrates this performance boost for the DPOTRF kernel on a two-dimensional geospatial covariance matrix represented to tolerance 10^−8^ or better in Froebenius norm for each tile for three generations of Intel manycore and two generations of algorithms, for a range of matrix sizes from 27 K up to 297 K, as memory capacity allows. The classical algorithm follows an *O*(*n*^3^) scaling and can be extended only up to matrices of dimension 108 K on an Intel Sandy Bridge processor using Intel’s Matrix Kernel Library (MKL) [18]. Successive generations of Intel processor hardware, namely Haswell and Skylake, provide runtime improvements (red arrows) and memory capacity improvements while following the same scaling. The TLR algorithm as implemented in the HiCMA Library [17] shows closer to quadratic scaling in runtime with problem size, with significantly greater problem-size accommodation and runtime reductions (green arrows) on the same hardware. The blue arrow shows the product of the hardware and algorithmic advances, already more than two orders of magnitude for matrices of dimension 108 K and growing with size. We focus on factorization time only since the time to generate and compress relative to the factorization time decreases due to the difference in the asymptotic complexities of the respective phases [19].

**Figure 4.**
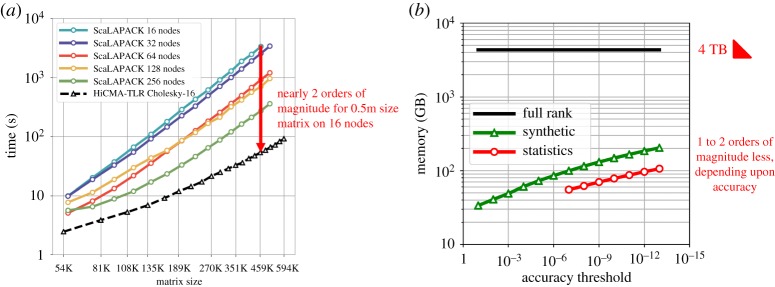
Distributed-memory implementations of DPOTRF on a Haswell-based Cray XC40, on 16 through 256 nodes for ScaLAPACK and TLR on 16 nodes (*a*). Memory footprints of double precision synthetic and covariance matrices of dimension 1M for a range of block tolerance thresholds, compared with fully dense (*b*). (Online version in colour.)

Still larger matrix sizes can be accommodated by distributed-memory versions of (P)DPOTRF and HiCMA, using MPI, as shown on figure 4*a*. With the memory savings of TLR, larger problems can be accommodated for a given node count, and nearly two orders of magnitude of runtime improvement come from algorithmic improvement, better than from the comparable ratios of concurrency. A quantification of the memory footprint improvement of TLR is shown on the right in figure 4. A symmetric matrix of dimension 1M stored in the lower triangle in 8-Byte precision requires 4 TB. With a tight tolerance of 10^−13^, worthy of essentially full precision, TLR enables more than an order of magnitude of storage savings. Depending upon the compressibility of the matrix and the accuracy threshold, nearly two orders of magnitude of storage savings are possible. The matrix is generated tile-by-tile using a user-defined matrix kernel and compressed on the fly. Therefore, at no single point in time does the full dense matrix need to reside in main memory.

**Figure 5.**
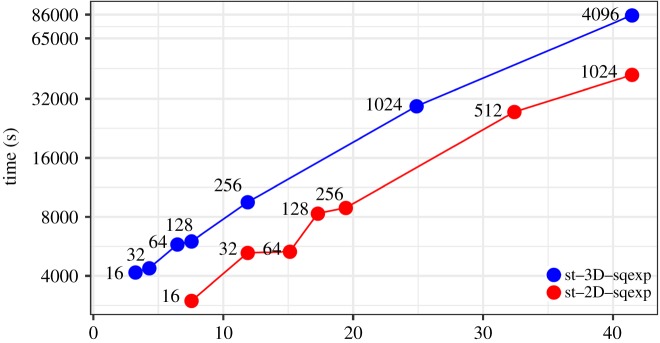
DPOTRF on a Haswell-based Cray XC40, on 16 through 4096 nodes for a custom distribution implementation, accommodating the largest matrix that can fit in each partition, up to dimension 42 million. (Online version in colour.)

Figure 5 is a *tour de force* Cholesky factorization of a TLR matrix derived from a Gaussian covariance of a random distribution of points in 3D, on up to 4096 nodes (131 072 cores). There is a larger discrepancy in ranks for tiles of uniform size when represented to uniform tolerance in 3D than in 2D, increasing the importance of DAG-based taskification. For distributed-memory aspects, it is also important to evenly distribute the ‘heavy’ diagonal tiles, which is not guaranteed by the standard two-dimensional block cyclic data distribution available in ScaLAPACK. Taking this matter into account with a heuristically derived process grid [20], we have factored a TLR covariance matrix of dimension 42 million (representing the covariances of this many points in a 3D cube) in less than 24 h. Notwithstanding that the dense covariance matrix would not fit in the available DRAM memory, if we extrapolate its solution time using the same peak computational rate of approximately 3.7 PFlop/s the conventional factorization would require 77 days. If DRAM is exhausted, out-of-core approaches may be considered. The StarPU dynamic runtime system provides support for out-of-core algorithms that can mitigate data motion overhead [21].

TLR is attractive because it can be retrofit into existing tile-based shared-memory and distributed-memory software by simply overloading the fully dense matrix kernel operations with their low-rank counterparts. However, it does not achieve storage or operational optimality. For memory-limited accelerators like GPUs further advances in the algorithm have been achieved and efficiently implemented, as in the next section.

## 3. Hierarchical low-rank representation

Fully hierarchical low-rank representations improve over TLR representations in storage and operational complexity at the cost of managing tree-like data structures and load balancing with variable block sizes. The apparent disadvantage of the latter is mitigated by the ability of some H-matrix formats to operate with much less variability in the effective rank of each block, which is a complementary spoiler of load balance. Hierarchical matrices shrink the memory footprints of matrices allowing the data to live higher on the memory hierarchy during operations. Furthermore, the algorithms at the core of hierarchical matrix operations can be written in terms of high arithmetic intensity GEMM, QR, randomized SVD, Cholesky factorization, and other dense linear algebraic operations that operate on large batches of small blocks and are able to take advantage of modern manycore architectures.

Hierarchical matrices, denoted by H [22] provide an approximate algebra in which tunable-accuracy approximations of certain classes of fully dense matrices can be stored and operated on in linear or log-linear space and time complexity. The tunable accuracy aspect of H-matrices is particularly valuable because it allows the ranks *k* of the matrix blocks to be controlled, and therefore the memory footprint of the matrix to be restricted. *k* grows as *O*(|log*ϵ*|^*d*+1^) for operators coming from elliptic boundary value problems in *d* dimensions [23], and this invites using loose *ϵ* to obtain low accuracy approximations that may, for example, be adequate as preconditioners [24].

### (a) Blocking and data structures

Hierarchical matrix representations may be classified along two axes: one describing the block structure of the matrix, and the other describing the form of the representation of the low-rank data within the blocks.

**Figure 6.**
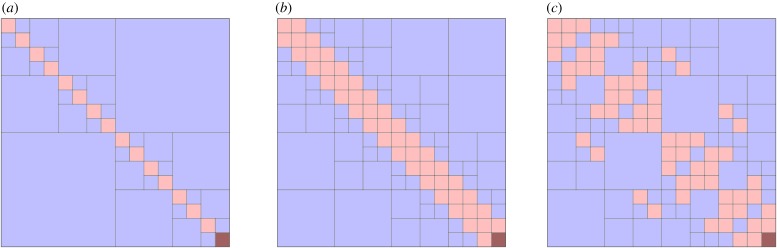
Hierarchical block structure for weak (*a*) and standard or strong admissibility (*b*,*c*). (Online version in colour.)

Figure 6 shows three sample blocking structures. The first structure, called a ‘weak-admissibility’ blocking, is perhaps the simplest, in which each off-diagonal low-rank block touches the diagonal. This simple subdivision of the blocks is convenient and attractive for implementation. But, not unexpectedly, it suffers from the fact that very large ranks may be needed to achieve acceptable accuracy for many applications, particularly in three-dimensional problems. ‘Standard-admissibility’ blocking, also called ‘strong-admissibility’ blocking, further refines the blocks using an admissibility criterion for determining whether a block should be further refined. When the matrix comes from a spatial discretization, the admissibility condition is naturally geometric in nature and depends on the separation between the cluster of nodes corresponding to the rows of the block and the cluster corresponding to the columns of the block. If these two clusters are sufficiently far away the interaction between them is smooth and therefore representable with a low-rank representation of bounded rank. Small blocks are stored in their dense form. In practice, block refinement stops at block sizes that are determined by the hardware and cache sizes. The middle and right matrix block structures of figure 6 come from different admissibility criteria. The middle matrix clusters nodes along a one-dimensional axis, which may be curved, and measures distance along that axis. This results in a structure where the blocks become increasingly larger away from the main diagonal. The right matrix illustrates the result of clustering the nodes using a general spatial partitioning strategy (kd-tree, segments of a space-filling curve, etc.) and using cartesian distance between the clusters for checking admissibility. This results in a more irregular structure. However, as it is able to refine the matrix in regions needed to capture non-smooth blocks, it can generally approximate the matrix with smaller ranks in the blocks. For a given target accuracy, there are compute/memory trade-offs between using fewer blocks of larger rank (using coarse admissibility parameter and getting closer to weak admissibility) or more blocks of smaller rank (standard admissibility with a tight admissibility parameter).

**Figure 7.**
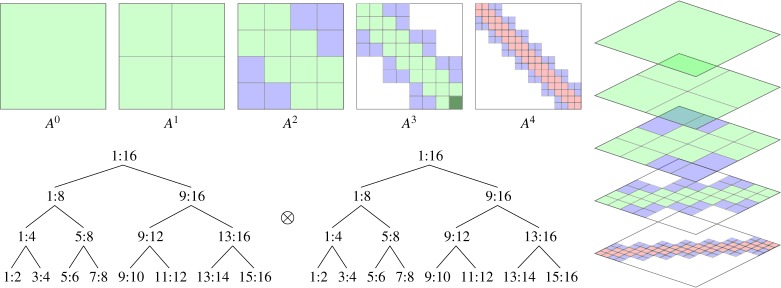
Recursive construction of an H-matrix. Starting from the top-level row and column cluster trees are dual traversed, checking if a matrix block is admissible as a low-rank block (blue) or needs to be further subdivided (green) with its children checked at the next level. At the finest level, small inadmissible blocks are stored as dense blocks (red). (Online version in colour.)

Figure 7 illustrates the construction of the block structure of a hierarchical matrix. Two trees are first generated by recursively subdividing the node sets corresponding to the rows and columns. For square matrices representing a complete operator, both rows and columns have the same tree structure. But as a purely algebraic matrix representation, these trees may be of different sizes (corresponding to rectangular hierarchical matrices) and may be subdivided differently with different cluster sizes at their leaves. After building these trees, they are traversed using a dual-traversal strategy similar to that used in FMM codes to decide at every level whether or not a matrix block is admissible and therefore representable as a low-rank one or that it is to be subdivided further and its children handled at the next level down in the tree traversal. The result of this step is an incomplete tree obtained as the cross product of the two cluster trees. When the row and column cluster trees are binary trees, this cross-product tree is a quadtree. Its leaves correspond to blocks of the matrix. For efficiency, particularly on GPUs, this tree is flattened and stored per-level with each block indexed by its Morton order index.

Hierarchical matrices are further classified based on the form of the numerical representation of the matrix block data. The simplest representation represents every block independently with its own low-rank factors $A_{ij}^{l}=U_{ij}^{l}{V_{ij}^{l}}^{T}$, where $A_{ij}^{l}$ is the block *ij* of level *l* and the *U* and *V* factor are rank *k* factors. This representation requires a storage cost *O*(*kn*log *n*), where *k* is a representative rank of the matrix blocks. It is possible to obtain an asymptotically optimal memory footprint *O*(*kn*) by using a more refined representation of the column and row bases of the blocks. In this representation, the column and row bases are common to all blocks of a given block row at level *l*, i.e. $A_{ij}^{l}=U_{i}^{l}S_{ij}^{l}{V_{j}^{l}}^{T}$, where $U_{i}^{l}$ is independent of *j* and every block is now representable by a small *k* × *k* coupling matrix $S_{ij}^{l}$ in these bases. In a final improvement, the basis matrices are nested, i.e. the basis $U_{i}^{l}$ of a block row is not explicitly stored, but generated on-demand from the bases of its children using the recursion $U_{i}^{l}=\sum_{c}U_{ic}^{l+1}E_{ic}^{l+1}$ which continues all the way to the leaves. Only the bases at the leaves, along with small transfer matrices $E_{ic}^{l}$, need to be stored, saving a log factor in storage. This representation, known as $H^{2}$ to denote the hierarchies in both the block structure and the low-rank bases, has the optimal memory footprint asymptotically. This latter property is the primary reason that makes $H^{2}$ the best match for GPUs which have rather austere memories.

### (b) Linear algebra operations with hierarchical matrices

As part of the HiCMA library, we have developed modules to support hierarchical matrices. The library runs on CPUs, where it uses OpenMP for parallelization, as well as on GPUs, where it uses batched kernels to allow it to deliver exceptional performance. We believe this is the first GPU library that supports the $H^{2}$ nested basis format with a general block structure of the matrix. In this section, we highlight the key algorithms and operations of the library and show representative performance results.

One key idea for obtaining performance is to cast the algorithms on level-by-level flattened trees and use batched dense linear algebra kernels (batched GEMV, GEMM, QR, Cholesky and SVD) on the operations at every level. Batched dense kernels operate on large batches of small blocks and achieve very high performance through their ability to place data high on the memory hierarchy in shared memory and registers when needed. This allows our library to achieve two essential desiderata: optimal asymptotic memory footprint as well as small constants in the runtime complexity. Using this design pattern, the apparent irregularity of the tree data structures of general hierarchical representations no longer impedes performance, as we illustrate below with matrix-vector and matrix-matrix operations. The basic implementation template divides operations into two phases. In a first phase, we ‘marshal’ the irregularly laid out data in a way that allows them to be used by the batched dense linear algebra routines. Marshaling operations only involve pointer arithmetic with no data movement and no floating-point operations, and as a result have minimal overhead. In a second phase, the batched kernels are executed on the floating-point data.

We first consider the hierarchical matrix-vector multiplication operation. The low-rank portion of the computation may be written as

$$Ax=\left(\sum_{l}\sum_{(i,j)\in L_{l}}U_{i}^{l}S_{ij}^{l}{V_{j}^{l}}^{T}\right)x$$

where *L*_*l*_ is the set of low-rank blocks at level *l*. The operation is performed in three steps: (1) the products of ${V_{j}^{l}}^{T}x$ are performed for all basis nodes *j* at all levels *l*. This is performed via an upsweep through the row cluster basis tree *V* with batched matrix-vector kernels at every level; (2) the products $S_{ij}^{l}$ with the vectors of step 1 can be performed for all blocks *ij* of all levels in parallel using batched matrix-vector kernels, as well; (3) finally, the product of $U_{i}^{l}$ with the vectors of step 2 may be obtained via a downsweep through the column basis tree, and accumulated. The products of the dense blocks of the matrix with the vector *x* can be performed separately and independently of the low-rank portion. By overlapping these dense products with the three steps above via streams, we are able to keep the GPU saturated, even when there is not enough work for all threads in the low-rank part at the top levels of the trees.

**Figure 8.**
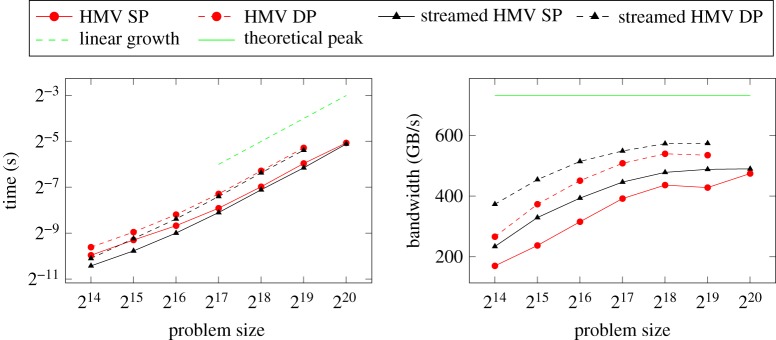
Linear temporal scaling and achieved memory bandwidth of hierarchical matrix-vector multiplication in single and double precision over a range of 3D covariance matrices performed to a target accuracy of 10^−3^, with (black) and without (red) streaming. (Online version in colour.)

Figure 8 shows the performance obtained on the P100 GPU for sample problems of increasing size resulting from a 3D covariance matrix with an exponentially decaying kernel. The algorithm shows an optimal *O*(*n*) asymptotic performance. Concurrent streaming of the low-rank blocks and dense blocks helps improve performance by hiding latencies but the effect is noticeable only on matrices of relatively small size where there is not enough work at the coarse tree levels. But the more important performance metric to note in this bandwidth-constrained computation is that it achieves 78% of the theoretical peak bandwidth of the GPU through its careful orchestration of data movement during its operations [25].

$H^{2}$ matrices may be regarded as the algebraic generalizations of FMM because the three-step structure of the matrix-vector operation with the low-rank portion of the matrix corresponds very closely to the computations of the weak interactions between particles in an FMM method. Particle to multipole and multipole to multipole interactions are the leaf level and the upsweep operations through the row basis tree *V*. Multipole to local expansions correspond to the multiplications with the blocks of the coupling tree *S* of the second step. Finally, local to local expansions and local to particle interactions are the downsweep and leaf level operations through the column basis tree *U* of the third step. While FMM computations are only possible when there is an analytical kernel that can be evaluated, hierarchical matrices, by explicitly storing matrix blocks algebraically, can naturally handle general dense problems and not restricted to matrices with an underlying analytical kernel.

**Figure 9.**
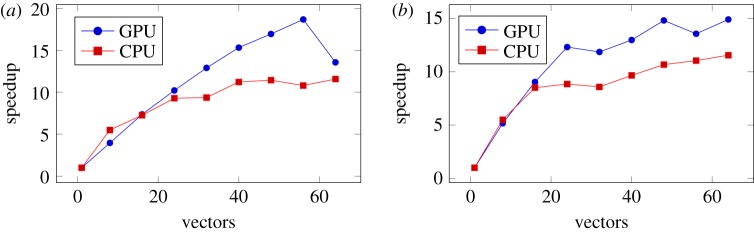
Speedup in the performance of the hierarchical matrix-vector multiplication operation with multiple vectors relative to a single vector on a problem of size *n* = 2^19^. (*a*) Single precision, (*b*) double precision. Dips in performance are due to jump increases in thread allocation in the batched kernels at certain discrete sizes, resulting in overallocation of resources. (Online version in colour.)

When performing matrix-vector multiplication with multiple vectors rather than a single one, the increased arithmetic intensity produces substantial performance benefits over single vector operations both on CPUs and GPUs as shown in figure 9. By replacing the batched dense matrix-vector routines by corresponding batched dense matrix matrix (GEMM) routines, the hierarchical matrix multiplication operation inherits all the performance benefits of the latter. This effect is more pronounced on GPUs because of their deeper memory hierarchies, and the high parallelism and coalesced memory accesses of the compute-bound GEMMs. In fact, the multiple vector operation can achieve a substantial portion (greater than 90%) of the batched GEMM performance, which on the P100 is about 1.6 TFLOP/s in double and 2.8 TFLOP/s in single precision.

Next, we consider a low-rank update operation, since a number of algorithms in the library use an operation of the form *A* = *A* + *XY*^*T*^ as a building block. The low-rank update could be a global update where *X* and *Y* are dense matrices of size *n* × *r*, or could be a set of local low-rank updates affecting only a portion of the matrix *A*, where rows of *X* and *Y* represent a subset of the *n* matrix rows. The first step in the low-rank update operations is to add the appropriate contributions of *XY*^*T*^ to the various matrix blocks at all levels. This of course increases the apparent rank of the blocks by *r*. The key task in executing the update efficiently is then to recompress the resulting matrix to the desired accuracy of the target approximation. This involves the generation of a new nested basis and compressing all blocks using it [25]. The key kernels involved in this operation involve downsweeps through the basis trees with QR factorization being performed. At every level, all low-rank blocks of a given block row/column are stacked and a QR factorization is performed. The *R* data generated is used in constructing the new basis for the resulting matrix. The compression of the basis involves an upsweep through the basis trees where at every level SVD factorizations are performed on stacks of small transfer matrices. Performance is obtained because the large amount of compute-intensive factorizations, both QR and SVD, that are performed at every level can be efficiently executed by batched kernels. We have developed batched QR and batched adaptive randomized SVD operations for this purpose [26,27].

**Figure 10.**
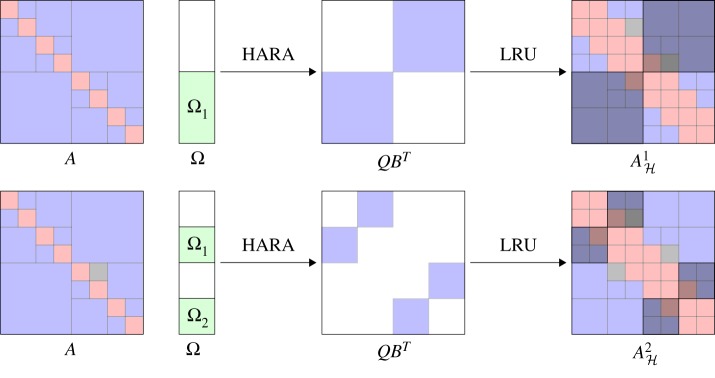
The major phases of one level of the hierarchical adaptive randomized approximation (HARA). The top left figure shows the matrix *A* that is being approximated with the leaves of the sampling tree overlaid on it, as well as the structured random vectors (green) generated by the sampling tree. These are used to generate the low-rank updates of the top centre figure. Finally, the updates are applied to the hierarchical matrix $A_{H}$ of the top right figure, where the affected leaves of the matrix tree are highlighted in the shaded areas. The bottom set shows the sampling of the second level of the matrix and updating the corresponding matrix blocks. (Online version in colour.)

We build on the hierarchical matrix vector and the low-rank update operations, to develop an algorithm for constructing a hierarchical matrix approximation of a ‘black box’ matrix that is accessible only via matrix-vector products [27]. The algorithm generalizes the popular randomized algorithms for generating low-rank approximations of large dense matrices [13] to the case of general hierarchical matrices $H^{2}$. Figure 10 shows the high-level structure of the algorithm, which operates by levels. At every level, blocks of the ‘black box’ matrix *A* are sampled by multiplying *A* with structured random vectors with patterns chosen to sample the desired blocks. Low-rank approximation of these blocks are turned into low-rank updates (LRU) to be added to the hierarchical matrix approximation being constructed. The sampling of blocks at lower levels of the hierarchal matrix tree is done by subtracting from the sampling of *A* the effect of the blocks from the higher levels just constructed. The process continues until the leaves are reached. The two main ingredients of the algorithm, beyond matvecs with *A*, are a hierarchical matrix-vector product for sampling and a low-rank update for repeated compression. Therefore, substantial performance is obtained due to the use of these operations as described earlier.

**Figure 11.**
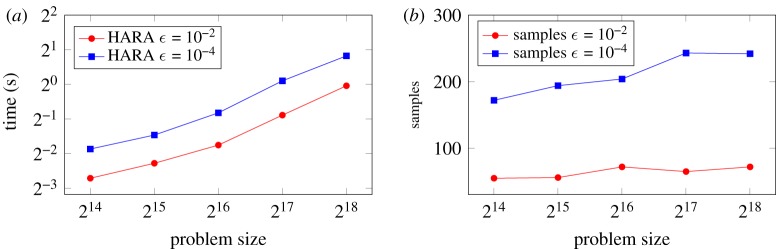
Time required for the multiplication of two square matrices (*a*) and number of samples required to construct the product as an $H^{2}$ matrix (*b*) to specified accuracies over a range of covariance matrices of increasing size. (Online version in colour.)

We can readily use the construction operation above to compute the hierarchical matrix resulting from a general matrix expression with hierarchical matrix operands. Consider, for example, a given hierarchical matrix *A* and the task of computing its square *B* = *A*^2^. The matrix *B* is accessible to us via matrix-vector products since we can evaluate the desired product using two fast hierarchical matrix-vector operations with *A*, *B x* = *A* (*Ax*) = *Az*. Armed with the ability to sample *B* in this fashion, we apply the randomized hierarchical construction algorithm to obtain *B* using a sequence of low-rank updates, level by level. Figure 11 shows the performance of this matrix-matrix operation performed to two target accuracies on a number of representative matrices. The number of samples needed to produce *B* is also shown. We observe a growth in runtime that is converging to the expected log-linear asymptotic complexity as the problem size increases, and note the absolute performance that allows the multiplication of two square matrices of size 256 K in under two seconds to moderate accuracy on a P100 GPU. Inverses, square roots, and factorizations of hierarchical matrices may also be obtained efficiently and are the focus of ongoing work.

## 4. Programming practices for exascale architectures

**Figure 12.**
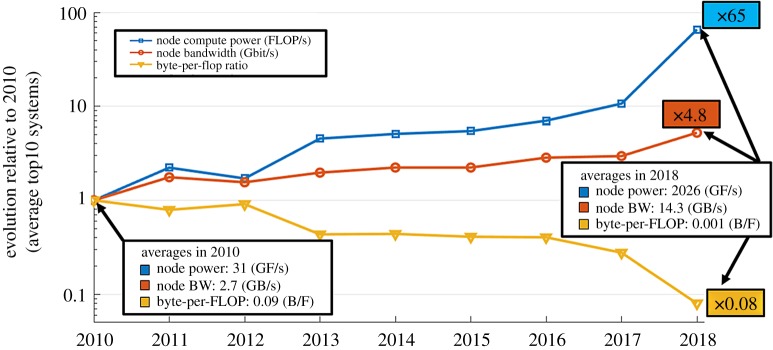
Deteriorating ratio of Bytes/Flop averaged over the top 10 ranked systems by the HPL benchmark (see [3]). (Online version in colour.)

Over the last decade, the theoretical peak flop/s rate of individual nodes of the highest performing computers has increased faster than the theoretical peak memory bandwidth, as illustrated in figure 12, which updates by two years the original figure in [3], resulting in more than an order of magnitude deterioration in their ratio. The #1 ranked system as of this writing, Summit at ORNL, can perform about 2000 flops in the time it takes to load a Byte, or about 16 000 flops in the time it takes to load a double-precision word. This is the primary reason that algorithms for exascale hardware must be redesigned to operate on operands high in the memory hierarchy, just cycles away, and the primary efficiency-driven reason to design data sparse kernels. Contemporary architectures charge exorbitant latency and energy penalties for pulling in data from afar. At the same time, the peak flop/s rate promised from exascale hardware is realizable only if SIMT-style vector and matrix operations can be used. At first glance, this introduces a tension between architecture and complex algorithms with tree-like data structures and scale recurrence. However, through a variety of techniques, this apparent mismatch can be spanned.

After decades of programming model stability with bulk synchronous processing, new programming models must be co-designed with the hardware. The fundamental tool is a dynamic runtime system that consumes a DAG whose vertices are operations, whose incoming edges are arguments required from previous operations, and whose outgoing edges are results for subsequent operations, as illustrated in figure 1. A DAG-based expression of a computation is usually superior to its expression in a compiled procedural language with loops and subroutine calls. The latter tends to restrict operations to a specific order. DAG-based dynamic runtime engines can remove artifactual synchronizations in the form of subroutine boundaries, remove artifactual orderings in the form of pre-scheduled loops, expose the native concurrency, and shorten the critical path. With sufficient cores, they can allow loops to be unrolled, whose different instances can overlap in time. Their disadvantages are the overhead of constructing and managing a task graph and the potential loss of memory locality to exploit the maximum concurrency when a core becomes idle and the nearest data is in a different memory basin. In practice, runtimes can be made NUMA-aware. Among the popular frameworks that embody this philosophy are Quark, StarPU, PaRSec, Charm++, Legion, OmpSs, HPX, ADLB and Argo.

Implemented with task-based dynamic runtime systems, hierarchical and tile-based algorithms expose concurrency at the block level, relaxing the synchrony of traditional full matrix algorithms with global loop limits. Task-based programming is even more important for energy-austere architectures, since there may be significant non-uniformity in processing rates of different cores even if task sizes can be controlled.

Exascale architectures emphasize SIMT-style programming at the node level, which can be catered to by batched operations on concurrently available small blocks. Tiling and recursive subdivision create large numbers of small problems that can be marshaled for batched operations on GPUs and MICs, amortizing kernel launch overheads. Performance programming calls for a polyalgorithmic approach that chooses different approaches for different block sizes [28]. To reduce sensitivity to the deep memory hierarchy and partially overcome the latency mismatch between even relatively fast layers of memory near the processing cores and the cores themselves, a variety of techniques can be employed including coalesced memory accesses, double-buffering, and non-temporal stores. The disadvantages are the complexity of the code and its architecture-specificity towards the bottom. The HiCMA library [17] is an interface to a multilayer programming system that tackles the high-performance challenges for a subset of linear algebra kernels of importance to our workload, when we find that these kernels are either missing from standard libraries, or that we have insight into how to make them perform better [12,26,29].

Some modules of HiCMA software, KBLAS [29] and KSVD [30] have been incorporated into the libraries of major vendors for by now several releases, respectively cuBLAS [31] and LibSci [32], and more modules are on the way, including all that were employed in creation of this article.

## 5. A hierarchical hourglass future

Two universes of computational linear algebra exist today side-by-side, a flat, sequential universe in which algorithms are simply stated with loops over global address spaces that typically process a row or a column at a time and another universe in which algorithms are restated for performance in ways that exploit hierarchy, with loops over local ranges only at each hierarchical level. The flat universe of computer architecture, dating to von Neumann, exists only in textbooks. We might prefer the simplicity of implementing hierarchical algorithms on flat-memory architectures, or, if we have access only to hierarchical memory architectures, of implementing flat memory algorithms upon them, but neither luxury exists. To better exploit emerging exascale architectures, we need new implementations of linear, least squares, eigenvalue, and singular value solvers that offer tunable accuracy-space-time trade-offs, that offer massive concurrency, reduce synchrony and over-specification of ordering, and dwell as high as possible on the memory hierarchy. We have shown herein how block structuring with a hierarchy of rank opens fresh means of achieving all of these goals.

It is worth connecting here to other hierarchies with roles to play in exploiting the exascale. It has long been recognized that a *hierarchy of discrete representations* of a single system at different levels of resolution can reduce overall complexity, obtaining a result worthy of the finest model while performing a majority of the work on coarser models. Multigrid [33] and Multilevel Monte Carlo [34] exemplify this philosophy. A *hierarchy of floating point precisions* has become available in recently released hardware and studies show how up to three precisions can be employed within a single task to produce results worthy of the highest precision while the computationally complex steps are performance in low precision [35]. The advantages in stretching the memory storage and bandwidth of a given architecture to accommodate larger problems should be sufficient to bring performance-oriented programmers over to the hierarchical precision universe. A *hierarchy of data types* is required to support this next generation of coding practices, beginning with precisions. A *hierarchy of reliabilities* for data retrieval and storage may also be built into systems beyond exascale. This topic is beyond the present scope other than to note that it is another type of hierarchy. Some data must be reliably stored and retrieved or the reliability of the overall code is compromised. Other data can tolerate (relatively rare) errors because such errors will be caught and corrected downstream. For instance, a reliable residual computation allows an iterative linear algebra code to recover from an erroneous preconditioner application or solution vector update. As higher premiums are placed on the energy efficiency there will be advantages in accepting small overheads of re-computation for large savings in the typical computation.

**Figure 13.**
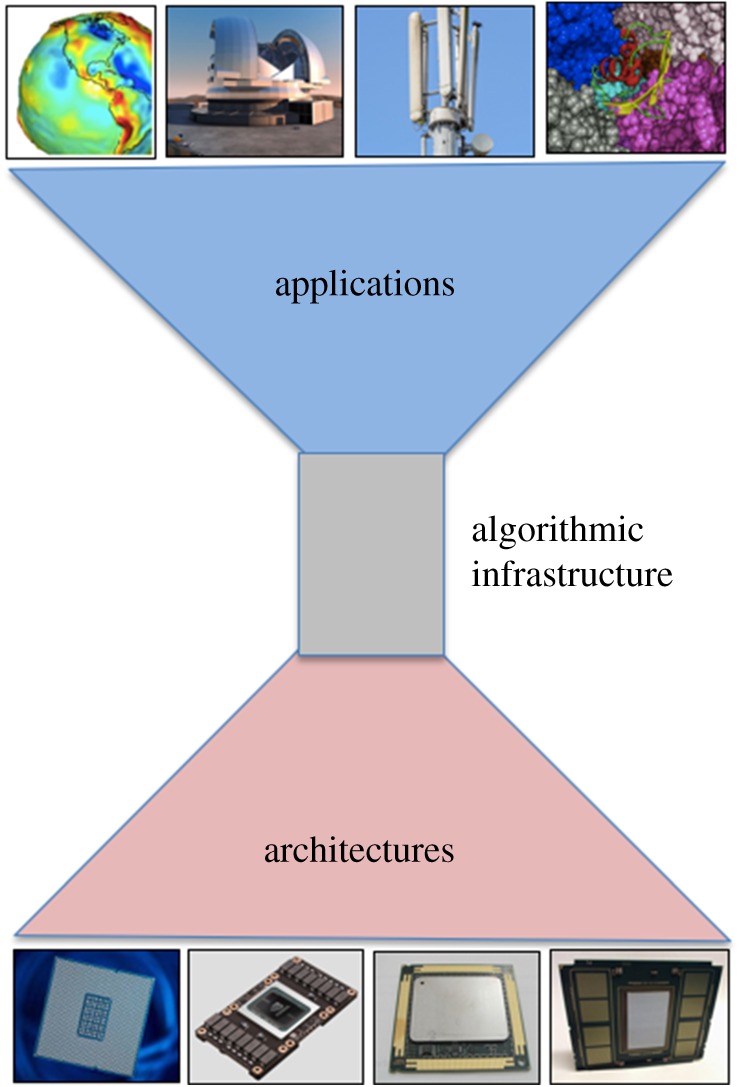
Hourglass model.

As hierarchical algorithms for hierarchical architectures make code more complex, the future of scientific software must have the ‘hourglass’ structure [36], whereby many application programmers can invoke core infrastructure such as linear algebra through a common API and run efficiently on a wide variety of architectures through tailoring efforts such as those described in §4 (figure 13). Plenty of ideas exist to adapt or substitute for favourite solvers with methods that have higher residence on the memory hierarchy, greater SIMT/SIMD-style shared-memory concurrency, and reduced synchrony in frequency and/or span. Programming models and runtimes may have to be stretched to accommodate, and everything should be on the table for efficiency trade-offs. We conclude with a paraphrase of Shakespeare (The Merry Wives of Windsor, Act II, Scene ii): ‘If you can speed up linear algebra kernels, the world’s your oyster, which you with sword will open’.

## Footnotes

1 HiCMA is a transliteration of the Arabic word for ‘wisdom’

## Data accessibility

HiCMA software modules are available at https://github.com/ecrc/hicma.
